# Perceived mental illness stigma and self stigma among persons treated for psychotic disorders

**DOI:** 10.1192/j.eurpsy.2024.1510

**Published:** 2024-08-27

**Authors:** A. Jambrosic Sakoman, T. Jendričko, D. Bošnjak Kuharić, A. Tomić

**Affiliations:** ^1^Department for Psychotic Disorders; ^2^Department for Psyhotherapy, University Psychiatric Hospital Vrapče, Zagreb, Croatia

## Abstract

**Introduction:**

Stigma related to mental health has serious impact on persons suffering from psychiatric disorders and on their families. Self-stigma occurs when people with mental health problems internalize public attitudes, negative beliefs and stereotypes associated with psychiatric disorders. Stigma and self-stigma can affect every aspect of life and result in discrimination, social exclusion, feelings of low self-esteem, shame, guilt, and can postpone seeking help.

**Objectives:**

To examine perceived stigma and self-stigma of people treated for psychotic disorders.

**Methods:**

We will include male and female patients older than 18 years of age, diagnosed with psychotic disorders, treated as outpatients. Assessment will include sociodemographic data, Internalized Stigma of Mental Illness Inventory – 9-item Version (ISMI-9) * to measure internalized stigma of mental illness, The perceived devaluation-discrimination (PDD) scale to measure perceived stigma, the World Health Organisation Quality of Life-BREF (WHOQOL-BREF) questionnaire, and Clinical Global Impression Scale (CGI).

**Results:**

We will analyse differences in ISMI and PDD scales in patients treated for psychotic disorders.

**Conclusions:**

Understanding self-stigma and societal stigma associated with mental health is crucial in creating programs aimed at well-being of persons treated for psychotic disorders.

**Disclosure of Interest:**

None Declared

